# Dimensions of manic symptoms in youth: psychosocial impairment and cognitive performance in the IMAGEN sample

**DOI:** 10.1111/jcpp.12255

**Published:** 2014-05-28

**Authors:** Argyris Stringaris, Natalie Castellanos-Ryan, Tobias Banaschewski, Gareth J Barker, Arun L Bokde, Uli Bromberg, Christian Büchel, Mira Fauth-Bühler, Herta Flor, Vincent Frouin, Juergen Gallinat, Hugh Garavan, Penny Gowland, Andreas Heinz, Bernd Itterman, Claire Lawrence, Frauke Nees, Marie-Laure Paillere-Martinot, Tomas Paus, Zdenka Pausova, Marcella Rietschel, Michael N Smolka, Gunter Schumann, Robert Goodman, Patricia Conrod

**Affiliations:** 1 Institute of Psychiatry, King's College LondonLondon, UK; 2 Department of Psychiatry, Centre de recherche du CHU Ste-Justine, Montral UniversityMontreal, QC, Canada; 3 Department of Child and Adolescent Psychiatry and Psychotherapy, Central Institute of Mental HealthMannheim, Germany; 4 Department of Cognitive and Clinical Neuroscience, Central Institute of Mental Health, Medical Faculty Mannheim, University of HeidelbergMannheim, Germany; 5 Institute of Neuroscience, School of Medicine, Trinity College DublinDublin, Ireland; 6 University Medical Centre Hamburg-EppendorfHamburg, Germany; 7 Neurospin, CEAParis, France; 8 Department of Psychiatry and Psychology, Campus Charite Mitte, Charite Universitatsmedizin BerlinBerlin, Germany; 9 Department of Psychiatry and Psychology, University of VermontBurlington, VT, USA; 10 School of Physics and Astronomy, University of NottinghamNottingham, UK; 11 Physikalisch-Technische Bundesanstalt (PTB)Berlin, Germany; 12 School of Psychology, University of NottinghamNottingham, UK; 13 Institut National de la Sante et de la Recherche Medicale, INSERM CEA Unit 1000 Imaging & Psychiatry, University Paris SudOrsay, France; 14 Rotman Research Institute, University of TorontoToronto, ON, Canada; 15 Montreal Neurological Institute, McGill UniversityMontreal, QC, Canada; 16 The Hospital for Sick Children, University of TorontoToronto, ON, Canada; 17 Department of Genetic Epidemiology, Central Institute of Mental Health, Medical Faculty Mannheim, University of HeidelbergMannheim, Germany; 18 Neuroimaging Centre, Department of Psychiatry and Psychotherapy, Dresden University of TechnologyDresden, Germany

**Keywords:** Mania, bipolar, intelligence, adolescents, creativity

## Abstract

**Background:**

It has been reported that mania may be associated with superior cognitive performance. In this study, we test the hypothesis that manic symptoms in youth separate along two correlated dimensions and that a symptom constellation of high energy and cheerfulness is associated with superior cognitive performance.

**Method:**

We studied 1755 participants of the IMAGEN study, of average age 14.4 years (*SD* = 0.43), 50.7% girls. Manic symptoms were assessed using the Development and Wellbeing Assessment by interviewing parents and young people. Cognition was assessed using the Wechsler Intelligence Scale For Children (WISC-IV) and a response inhibition task.

**Results:**

Manic symptoms in youth formed two correlated dimensions: one termed *exuberance*, characterized by high energy and cheerfulness and one of *undercontrol* with distractibility, irritability and risk-taking behavior. Only the undercontrol, but not the exuberant dimension, was independently associated with measures of psychosocial impairment. In multivariate regression models, the exuberant, but not the undercontrolled, dimension was positively and significantly associated with verbal IQ by both parent- and self-report; conversely, the undercontrolled, but not the exuberant, dimension was associated with poor performance in a response inhibition task.

**Conclusions:**

Our findings suggest that manic symptoms in youth may form dimensions with distinct correlates. The results are in keeping with previous findings about superior performance associated with mania. Further research is required to study etiological differences between these symptom dimensions and their implications for clinical practice.

## Introduction

Bipolar disorder (BD) is a potentially lethal condition that can be associated with lifelong impairment and distress (Baldessarini & Tondo, 2003; Judd et al., 2005). Yet, people with BD are also said to show better than average outcomes in creativity and educational attainment. Indeed, it is a long- and widely held notion that BD, a serious mental illness, may also confer advantages to those who have it (Goodwin & Jamison, 2007) and there are several studies that suggest links between creativity and BD (Hurlow & MacCabe, 2011). Pioneering evidence in this field came from research into biographical records (Jamison, 1989) and selected populations (Andreasen, 1987; Andreasen & Glick, 1988; Andreasen & Powers, 1975; Jamison, 1989; Ludwig, 1994) (such as writers and artists). Such studies are, however, liable to selection and information biases. Nonetheless, in an epidemiological sample, which by design is not subject to such biases, it was also found that many more authors and artists were affected by BD compared to the population base rate (Kyaga et al., 2011). As creativity is difficult to operationalize (Hurlow & MacCabe, 2011), it is important to note that similarly paradoxical findings of superior achievements of people with BD are also found using outcomes that are easier to define. In the Dunedin cohort, participants with a lifetime history of mania stood out from patients with other psychiatric diagnoses by their higher than average IQ scores (Koenen et al., 2009); however, there were only a few participants with mania (*n* = 8) limiting the inferences that can be drawn. More recently, MacCabe et al. (2010) using a large Swedish national cohort study discovered a U-shaped relationship between school performance and BD: adolescents with either excellent or very poor school performance carried the risk for bipolar disorder in later life. By contrast, the relationship between school performance and schizophrenia was linear – low school grades conferred a higher risk (MacCabe et al., 2010).

Several hypotheses have been proposed to explain the link between BD and superior outcomes (Goodwin & Jamison, 2007). A broad set of explanations suggests that behaviors or symptoms, such as exuberance or positive affect more generally, may underlie creativity. For example, experimental evidence suggests that positive affect improves performance in tests of word associations (Isen, Johnson, Mertz, & Robinson, 1985). Additionally, a study of inpatients shows that full scale IQ scores may improve slightly during hypomania (Donnelly, Murphy, Goodwin, & Waldman, 1982). These studies therefore raise the question whether certain manic symptoms are more strongly linked with positive outcomes than others. Symptom heterogeneity has been proposed to explain differential etiological processes and outcomes in a number of disorders (Coghill & Sonuga-Barke, 2012). It is possible that our findings about two distinguishable dimensions of mania could underlie differences in mechanisms and clinical outcomes.

Recently, we have shown that manic symptoms in children and adolescents can be separated along two correlated dimensions, one of episodic undercontrol and one of episodic exuberance (Stringaris, Stahl, Santosh, & Goodman, 2011). Symptoms of the undercontrol dimension include distractibility, irritability, and poor inhibition, whereas high energy levels, cheerfulness, and a heightened sense of achievement were part of the exuberance dimension. Symptoms of undercontrol are strongly correlated with psychopathology, particularly attention deficit hyperactivity disorder (ADHD) and disruptive behavior disorders, whereas symptoms of exuberance are not associated with other psychopathology once symptoms of undercontrol have been taken into account (Stringaris et al., 2011). In addition, undercontrol symptoms are significant predictors of social impairment independently of the presence of other psychopathology (Stringaris et al., 2011). Similar findings have been obtained by others investigating the structure of manic symptoms in adolescents and adults. Holtmann et al. (2009) distinguished between ‘active-elated’ symptoms that were associated with good psychosocial adjustment, in contrast to dimensions of ‘irritable-erratic’ and ‘disinhibited’ symptoms that were related to disruptive behavior disorders in adolescents. Likewise, young adults scoring high on an ‘active-elated’ dimension of symptoms also scored high on measures of wellbeing, whereas those scoring high on ‘irritable/risk-taking dimensions’ experienced psychopathology and distress (Brand, Gerber, Puhse, & Holsboer-Trachsler, 2011). In an epidemiological study of adults with pure hypomania, high energy levels and positive affect were particularly common. Importantly, those with pure hypomania had higher incomes and were more likely to be married and reports less distress than controls (Gamma, Angst, Ajdacic-Gross, & Rossler, 2008).

These findings point to a possible heterogeneity within manic symptoms that could explain the apparent paradox of a devastating illness that is also associated with high achievements. Establishing such heterogeneity would carry important implications for understanding the pathophysiology of BD.

For this purpose, we use a dimensional approach in a community-based sample. We adopt a dimensional approach to manic symptoms, rather than a diagnostic approach. This is in keeping with the approach promoted by the National Institute of Health, Research Domain Criteria (RDoC) (Morris and Cuthbert, 2012). In this approach, domains or constructs such as positive valence or frustrative nonreward are probed in their relationship with different units of analysis, such as cognitive circuits or physiology. This is intended to remove the constraints of typically arbitrary diagnostic boundaries and help bridge the gap between clinical phenotypes and underlying mechanisms (Insel et al., 2010). It should be clear that the extent to which manic symptoms are on a continuum with bipolar disorder is an outstanding empirical question. It has been shown in US clinic samples that a high rate of manic symptoms in adolescents – typically falling short of meeting BD diagnostic criteria due to shorter duration – increases risk for subsequent BD (Birmaher et al., 2009). In addition, subthreshold manic symptoms in adolescents are associated with white-matters microstructure abnormalities in brain regions involved in mood disorders (Paillère Martinot et al., 2014). However, subthreshold manic symptoms have not been shown to predict later bipolar disorder in a Danish cohort (Päären et al., 2013). In this paper, we use a community-based sample; we avoid referral and other selection biases. In addition, we use easy to operationalize outcomes, namely intelligence and cognitive inhibition. Also, we test the robustness of our findings by analyzing first using self-report and then confirm by using parent-report and we test hypotheses about the relationship between dimensions of manic symptoms and cognitive outcomes. We use a large community-based sample of adolescents. There are several reasons to focus on this young age group. First, around half of adults with bipolar disorder report onset of their problems in childhood and adolescence (Perlis et al., 2009), yet we still know little about the structure and correlates of manic symptoms in youth. Research into early manic symptoms will be important for early recognition and prevention. Second, from an aetiological perspective, it is important to examine mechanisms of early manic symptoms. Recent results suggest that there may be developmental moderation in the pathomechanisms of bipolar disorder, such that for example deficits in response inhibition may be due to different mechanisms in adolescents than in adults (Weathers et al., 2012). Third, while there has been increasing research into bipolar disorder in youth, this is the first study to examine heterogeneity within manic symptoms and how this relates to cognition.

We test three hypotheses:

1. First, in keeping with previous work (Brand et al., 2011; Holtmann et al., 2009; Stringaris et al., 2011) we expect that manic symptoms in adolescents can be parsed into two correlated dimensions: one of undercontrol, characterized by irritable and disinhibited symptoms that is associated with psychosocial impairment, and an exuberant dimension that does not contribute independently to psychosocial impairment.
2. Second, we hypothesize that symptoms of exuberance will be positively associated with IQ scores, whereas symptoms of undercontrol will be negatively correlated with IQ. We expect that the exuberant dimension will be specifically associated with verbal IQ but not performance IQ, in keeping with suggestions about superior verbal performance in mania (Hurlow & MacCabe, 2011). To ensure that our findings are specific, we will also adjust for personality characteristics, such as sensation seeking or extraversion, as these have been suggested to correlate positively with intelligence in young people (Raine, Reynolds, Venables, & Mednick, 2002) and can also be related to manic symptoms (Bagby et al., 1997).
3. Third, we expect that only symptoms of undercontrol, but not of exuberance, will be associated with measures of poor response inhibition as has been previously described for bipolar disorder (Bora, Yucel, & Pantelis, 2009). To ensure that this association is specific for manic symptoms, we will also control for hyperactivity or conduct problems, as these are known to be associated with poor inhibition in young people.

## Methods

### Sample and procedure

A community-based sample of young adolescents (*N* = 2000) and their parents were recruited via high-schools in eight European sites as part of the IMAGEN Study (Schumann et al., 2010). The geographical areas were selected to maximize ethnic homogeneity (in light of the genetic component of the IMAGEN Study). However, private and state-funded schools were targeted in order to obtain a diverse sample of socioeconomic status, emotional and cognitive development. Parents gave informed written consent and adolescent's written assent. Local institutional ethics committees approved all study procedures. The IMAGEN study included a home assessment using an online computer platform and one or two study-center visits. Complete and reliable data sets of personality, psychiatric symptoms, IQ, and cognitive variables were available for 1755 participants (average age of 14.4 years (*SD* = 0.43), 50.7% girls.

### Measures

#### Development and well-being assessment (DAWBA)

The DAWBA (Ford, Goodman, & Meltzer, 2003) is a structured interview that also records verbatim accounts of problems. The questions are closely related to DSM-IV(APA, 2000) diagnostic criteria and focus on current problems. Adolescents were assigned a diagnosis only if their symptoms were causing significant distress or social impairment. The DAWBA interview was administered to all parents and to all adolescents. Manic symptoms were assessed as previously described (Stringaris et al., 2011) and as recently used to demonstrate white-matter abnormalities in subthreshold adolescent bipolarity (Leboyer, Henry, Paillere-Martinot, & Bellivier, 2005): The parents of adolescents themselves were presented with the following preamble: ‘Some young people have episodes of going abnormally high. During these episodes they can be unusually cheerful, full of energy, speeded up, talking fast, doing a lot, joking around, and needing less sleep. These episodes stand out because the young person is different from their normal self.' They were then asked: ‘Do you [Does X] ever go abnormally high?’, to which they had the options of answering: ‘No’, ‘A little’, ‘A lot’. For this screening question, answering ‘A little’ was associated with significantly more comorbidity and social impairment than answering ‘No’. In the interest of having as broad as possible a representation of subjects, we chose to include as ‘screen positive’ those who answered ‘A little’ as well as those who answered ‘A lot’—enquiring whether 26 specific symptoms of mania (including those stipulated by DSM-IV(APA, 2000) occurred during such episodes of going high. For each individual symptom, the participants had the option of choosing between one of the following answers: ‘No’, ‘A little’, ‘A lot’. The complete bipolar section of the interview can be seen at http://www.dawba.info/Bipolar/.

#### Strengths and difficulties questionnaire (SDQ)

We administered the parent- and youth-rated version of the SDQ, a questionnaire with robust psychometric properties (Goodman, 2001) that separately inquires about symptoms (hyperactivity/inattention; behavior problems; emotional symptoms; and peer problems) and impact in the form of psychosocial impairment (Stringaris & Goodman, 2013).

#### Personality

Extraversion was assessed using the NEO-Five Factor Inventory (Costa & McCrae, 1992) and sensation seeking was assessed using the Substance Use Risk Profile Scale (Woicik, Stewart, Pihl, & Conrod, 2009), as previously described (Castellanos-Ryan, O'Leary-Barrett, Sully, & Conrod, 2013).

#### Intelligence

Estimates of intelligence were derived from several subtests of the Wechsler Intelligence Scale for Children – Fourth Edition [WISC-IV; Wechsler, 2003 (Wechsler, 2003)]. To estimate verbal and spatial IQ the vocabulary, similarities, matrix reasoning and block design subtests were used. Verbal and Spatial IQ scores were then standardized by age.

#### Response inhibition

Response inhibition was assessed with a Go – no-Go task (Newman & Kosson, 1986). Participants learn by trial and error to respond to active stimuli or ‘good’ numbers for monetary reward and withhold response to passive stimuli or ‘bad’ numbers to avoid punishment (loss of previously gained rewards). Participants completed three conditions: a mixed condition of ‘punishment-reward’, a ‘punishment’ condition, and a ‘reward’ condition. The main dependent measure for this task was commission errors (i.e. failures to inhibit responses to passive stimuli) averaged across conditions, indicating a general deficit in response inhibition (Patterson & Newman, 1993). Average commission errors across conditions, as opposed to three separate condition-dependent measures, were used because commission errors across conditions were well correlated (*r* ≥ .50) and we had no specific hypotheses regarding reward or punishment sensitivity affecting response inhibition.

### Statistical analysis

#### Analytic strategy

To ensure that our findings are not due to specific informant effects, we conducted all our analyses for self-report and repeated them using parent-report.

To test our first hypothesis, we used a Confirmatory factorial analyses (CFA) to compare two alternative models: a one-factor solution where all symptoms of mania are part of a single dimension of symptoms; a two-factor model with two correlated but separable dimensions, one of undercontrol and one of exuberance, as previously described (Stringaris et al., 2011). Goodness-of-fit was compared on the basis of the Akaike information Criteria (AIC) and Bayesian information Criteria (BIC)-on both indices lower values indicate better fit. In addition, we tested our first hypothesis in a structural equation model with the SDQ score of the impact supplement (psychosocial impairment) as a dependent variable and the two latent dimensions of manic symptoms (undercontrol and exuberance) as predictors.

To test our second hypothesis, we used a structural equation model with either verbal IQ or performance IQ as the dependent variable and the two latent dimensions of manic symptoms (undercontrol and exuberance) as predictors. To test our prediction of specificity, we also ran the same models with verbal IQ as the outcome adjusting for performance IQ, and vice versa (with performance IQ as the outcome adjusting for verbal IQ). To ensure that our findings were not better explained by underlying personality dimensions, we also ran models with IQ as the dependent variable and the two latent dimensions of manic symptoms (undercontrol and exuberance) as predictors adjusted for either sensation seeking or extroversion. As a sensitivity test that is also free of distributional assumptions, we have repeated analyses for the main results of this paper using bootstrap estimation (20,000 iterations) and obtained a similar pattern of results.

To test our third hypothesis, we used a structural equation model with commission errors on the response inhibition task as the dependent variable and the two latent dimensions of manic symptoms (undercontrol and exuberance) as predictors. To test our prediction of specificity, we also we ran the same models with commission errors as the outcome adjusting for hyperactivity and conduct problems. All these analyses were controlled for sex (see below) and recruitment Center, as some of the outcome variables varied according to the site of data collection.

#### Missing values

After passing the screen question about episodic changes in mood described above, young people or their parents went on to respond to questions about manic symptoms: according to self-report: *n* = 986, parent-report: *n* = 699, or both: *n* = 440 (see online supporting information, Table S1 for demographic characteristics and comorbidities of the sample). For the analyses on verbal and performance IQ, data were available on *n* = 919 (93.2%) by self-report and *n* = 663 (94.8%) by parent-report. For the analyses on commissioning errors, data were available on *n* = 951 (96.5%) by self-report and *n* = 663 (96.3%) by parent-report. We present analyses using full-information maximum likelihood as implemented in MPlus version 5.32 (Muthén & Muthén, Los Angeles, CA). The pattern of results did not differ when analyses were done using list wise deletion approach in STATA, Version 11.2 (Stata Corp, College Drive, TX).

## Results

Our first hypothesis was that manic symptoms in adolescents could be parsed into two correlated dimensions each with different associations to psychosocial impairment. Indeed, a two-factor solution was preferable over a one-factor solution by self as well as by parent-report, on the basis of information criteria (*self-report*: two-factor solution AIC = 34,614, BIC = 34,898; *self-report*: one-factor solution AIC = 34,960, BIC = 35,239), (*parent-report*: two-factor solution AIC = 26,081, BIC = 26,385; *parent-report* one-factor solution AIC = 26,521, BIC = 26,820).

Table1 shows the frequency of each item for the two dimensions by parent- and self-report. Only items that have previously been shown to be associated reliably with each informant are shown; therefore some items do not overlap between informants. Overall, items in the exuberant dimension were more common than items in the undercontrol dimension by either reporting source and adolescents endorsed symptoms more frequently than did parents about their children. Note that the over-sexed item fit with other exuberant items by self-report, but with other undercontrol items by parent-report.

**Table 1:** Item frequencies and factor loadings for the two dimensions by self- and parent-report

	Self-reported manic symptoms *n* = 986		Parent-reported manic symptoms *n* = 699	
	(%)	Item loading	(%)	Item loading
**Exuberant**				
Cheerful	45.0	0.55	27.0	0.58
Fast talk	–	–	29.2	0.61
Active	45.5	0.60	26.2	0.65
Over-sexed	6.7	0.33	–	–
Gets more done	–	–	17.7	0.50
Noisy	29.3	0.58	26.9	0.55
Full of energy	55.3	0.56	35.9	0.54
Excitable	22.5	0.49	18.7	0.47
Joking	53.5	0.68	31.6	0.61
Outgoing	39.5	0.70	26.5	0.60
**Undercontrol**				
Restless	36.6	0.59	–	–
Over-sexed	–	–	2.4	0.42
Not concerned	20.0	0.54	–	–
Constant changes	–	–	4.3	0.60
Talks to strangers	–	–	5.3	0.39
Invades space	6.9	0.58	12.0	0.64
Overconfident	9.1	0.50	13.0	0.60
Risk taking	11.0	0.50	5.01	0.55
Irritable	4.5	0.33	9.3	0.53
Distractible	16.1	0.57	13.7	0.61
Less self-control	15.9	0.52	13.6	0.60
Poor concentration	5.1	0.59	13.4	0.67
Bossy	5.1	0.34	12.5	0.54
Unkempt	–	–	3.0	0.35
Flight of ideas	14.5	0.44	11.9	0.56

Because self-reported exuberance was higher in girls than boys (boys = 8.77, *SD* boys = 3.63; girls = 9.31, *SD* girls = 3.41; *t* = 2.50, *df* (984), *p* < .05) and parent-reported undercontrol higher in boys than girls (boys = 5.87, *SD* boys = 4.51; girls = 5.87, *SD* girls = 4.51; *t* = 3.30, *df* (697), *p* < .05), we adjusted for sex in all subsequent analyses. Age was not significantly associated with any of the dimensions (all *p* > .20; data available upon request).

In keeping with our first hypothesis, the undercontrol but not the exuberant dimension was significantly and positively associated with psycho-social impairment as judged by self-report on the SDQ impact score (*self-report*: undercontrol *β* = .28, *SE* = 0.05, *p* < .001; exuberance *β* = −.01, *SE* = 0.05, *p* > .5). By parent-report, only the undercontrol dimension was positively and significantly associated with impact, whereas the exuberant dimension had a weakly negative relation with impact (undercontrol *β* = .43, *SE* = 0.06, *p* < .001; exuberance *β* = −.12, *SE* = 0.06, *p* < .05).

**Figure 1:**
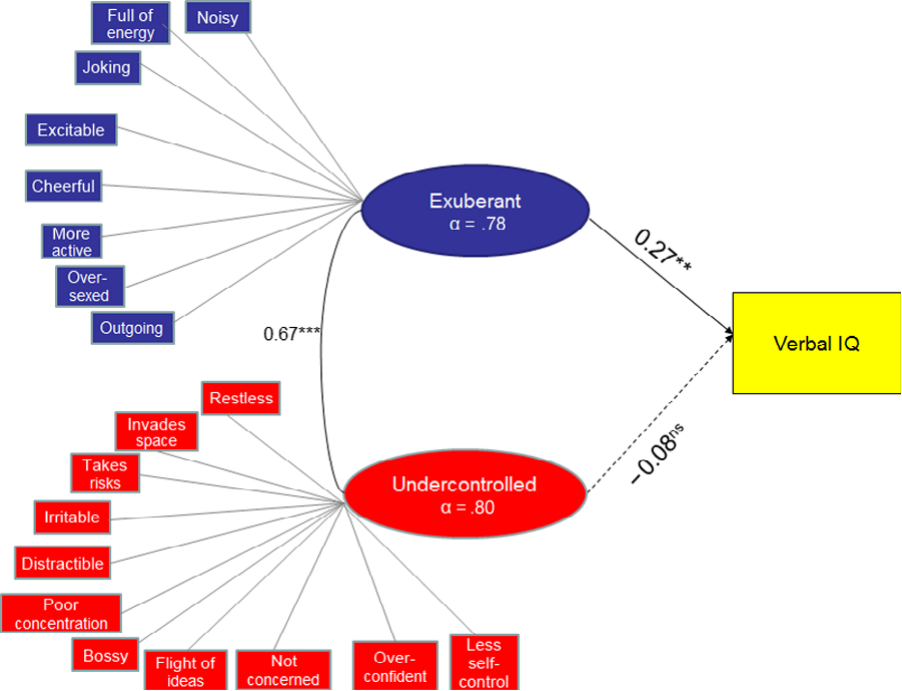
Self-reported manic symptoms and verbal IQ Results from a path analysis are shown for self-report, depicting the factor structure of mania symptoms on the left-hand side and the association with verbal IQ on the right. Squares represent individual mania symptoms or verbal IQ, latent mania dimensions are presented as ellipses. Standardized coefficients are presented for gender- and imaging center-adjusted models. *α* = Cronbach's alpha coefficient of internal consistency; ***p < 0.001; **p < 0.01; *p < 0.05; ns = non significant at *p* < 0.05

**Figure 2:**
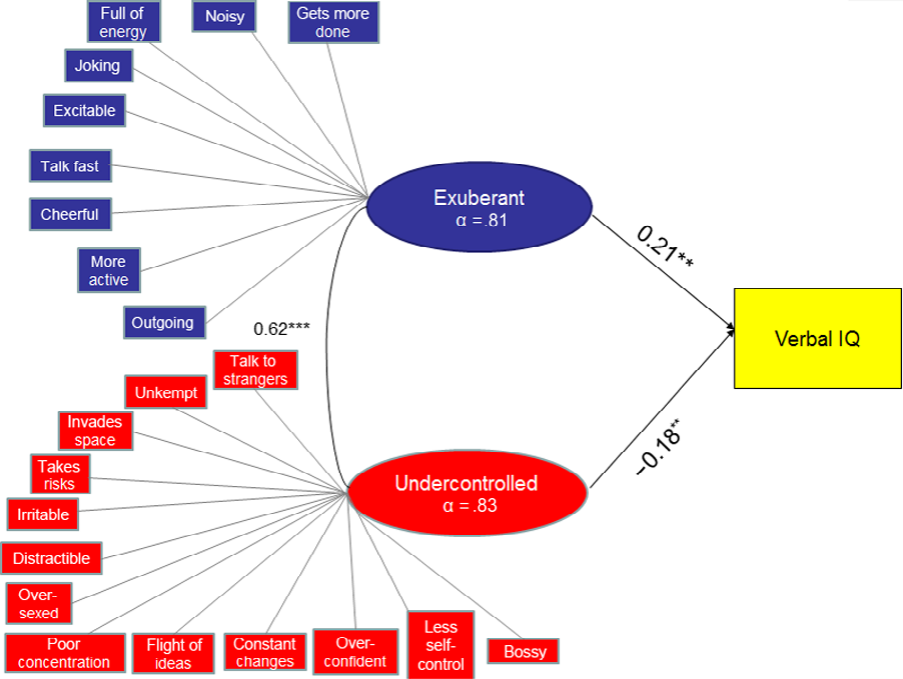
Parent-reported manic symptoms and verbal IQ Results from a path analysis are shown for parent-report, depicting the factor structure of mania symptoms on the left-hand side and the association with verbal IQ on the right. Squares represent individual mania symptoms or verbal IQ, latent mania dimensions are presented as ellipses. Standardized coefficients are presented for gender- and imaging center-adjusted models. *α* = Cronbach's coefficient of internal consistency; ***p < 0.001; **p < 0.01; *p < 0.05; ns = non significant at *p* < 0.05

Our second hypothesis was that exuberance would be positively associated with verbal IQ score, whereas undercontrol would be negatively correlated with verbal IQ. Figures1 and 2 present the relationship between the two dimensions of manic symptoms and verbal IQ as the outcome. As shown in Figures1 and 2, self- and parent-reported exuberance was positively associated with verbal IQ; conversely, undercontrol was negatively associated with verbal IQ only by parent but not by self-report. Table2 shows that exuberance was associated with performance IQ in simple models, but the association became nonsignificant after adjusting for verbal IQ. By contrast, exuberance was significantly associated with verbal IQ even after adjusting for performance IQ.

**Table 2:** Association of verbal and performance IQ with the exuberant and undercontrol dimensions

	Verbal IQ	Performance IQ	Verbal IQ adjusted for performance IQ	Performance IQ adjusted for verbal IQ	Verbal IQ adjusted for sensation seeking	Verbal IQ adjusted for extraversion
Self (*n* = 919)						
Exuberance	0.27^*^^*^^*^ (0.06)	0.20^*^^*^ (0.06)	0.18^*^^*^ (0.06)	0.09^ns^ (0.06)	0.26^*^^*^^*^ (0.06)	0.28^*^^*^^*^ (0.06)
Undercontrol	−0.08^ns^ (0.06)	−0.16^*^^*^ (0.06)	−0.01^ns^ (0.05)	−0.01^ns^ (0.05)	−0.08^ns^ (0.06)	−0.08^ns^ (0.06)
Parent (*n* = 663)						
Exuberance	0.21^*^^*^ (0.06)	0.18^*^^*^ (0.07)	0.13^*^ (0.06)	0.09^ns^ (0.06)	0.20^*^^*^ (0.06)	0.20^*^^*^ (0.06)
Undercontrol	−0.18^*^^*^ (0.06)	−0.22^*^^*^^*^ (0.06)	−0.09^ns^ (0.06)	−0.14^*^ (0.06)	−0.19^*^^*^ (0.06)	−0.18^*^^*^ (0.06)

Standardized (beta) regression coefficients and their standard errors are presented from gender-adjusted models with either verbal IQ or performance IQ as outcomes, unadjusted or adjusted for sensation seeking with exuberance and undercontrol as predictors. ^*^^*^^*^p < 0.001; ^*^^*^p < 0.01; ^*^p < 0.05; ns = non significant at *p* < 0.05.

Adjusting for the personality characteristics of sensation seeking and extraversion did not impact the association between the two dimensions of manic symptoms and verbal IQ, as shown in Table2.

**Figure 3:**
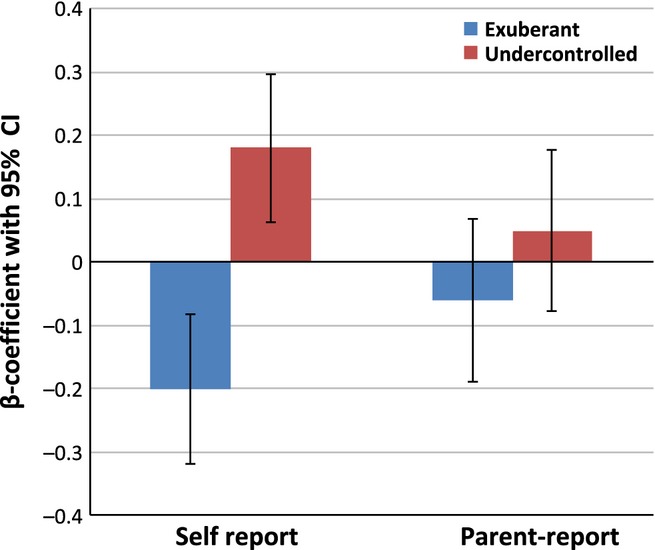
Association of commission errors with the exuberant and undercontrol dimensions. Results from a path analysis are shown for self- and parent-report. Standardized coefficients are presented for gender- and imaging center-adjusted models

Our third hypothesis was that undercontrol but not exuberance would be associated with measures of response inhibition. As illustrated in Figure3, self-reported undercontrol was positively associated with commission errors in a response inhibition task (i.e. pressing a button in no-go trials), whereas exuberance was negatively associated with commission errors. By parent-report, the associations went in the same direction, but were not statistically significant, as shown in Table3. The association between self-reported undercontrol and commission errors was not confounded by hyperactivity or conduct problems: Table3 shows that even after adjusting for hyperactivity and conduct scores, self-reported undercontrol was positively and significantly associated with commission errors, whereas exuberance was negatively and significantly associated with commission errors.

**Table 3:** Association of commission errors with the exuberant and undercontrol dimensions

	Commission errors	Commission errors adjusted for hyperactivity and conduct symptoms
Self (*n* = 951)		
Exuberance	−.20^*^^*^^*^ (.06)	−.19^*^^*^ (.07)
Undercontrol	.18^*^^*^ (.06)	.16^*^^*^ (.08)
Parent (*n* = 663)		
Exuberance	−.6^ns^ (.07)	−.7^ns^ (.07)
Undercontrol	.5^ns^ (.07)	.07^ns^ (.09)

Standardized (beta) regression coefficients and their standard errors are presented from gender-adjusted models with either commission errors, or commission errors adjusted for hyperactivity and conduct symptoms as the outcomes with exuberance and undercontrol as the predictor variables. ns = non significant at *p* < 0.05; ^*^p < 0.05; ^*^^*^p < 0.01; ^*^^*^^*^p < 0.001.

## Discussion

We used a general population sample to study the associations between manic symptoms in young people and cognitive performance.

In keeping with our first hypothesis, we found that manic symptoms in youth, can be parsed into two correlated dimensions (Stringaris et al., 2011): one of episodic undercontrol, characterized by irritable and disinhibited symptoms that is associated with social role impairment, and one of episodic exuberance that does not contribute independently to impairment. This is consistent with data from another general population sample (Stringaris et al., 2011). We also found that such manic symptoms are common in the general population of young people, although cases of bipolar disorder were rare (at or <0.1%), as shown in large population-based studies in the United States (Costello, Angold, & Keeler, 1999) and United Kingdom (Stringaris, Santosh, Leibenluft, & Goodman, 2010).

Consistent with our second hypothesis, we found that the exuberant, but not the undercontrolled, dimension was positively and specifically associated with verbal intelligence. This finding is in keeping with suggestions about superior verbal performance in mania (Hurlow & MacCabe, 2011). We also showed that this association could not be explained by other personality characteristics, namely sensation seeking and extroversion. However, we note that next to accumulating evidence about superior cognitive performance in people at risk for BD (Hurlow & MacCabe, 2011), there are also studies that show no evidence of such an association (Zammit et al., 2004).

As predicted in our third hypothesis, symptoms of undercontrol but not of exuberance were associated with measures of poor inhibition, as has been previously described for bipolar disorder (Bora et al., 2009); this association was not mediated by coexisting conduct or hyperactivity symptoms.

Our findings have several research and clinical implications. It is recognized that childhood conditions can encompass correlated dimensions, as in autism spectrum disorder (Ronald et al., 2006) and oppositionality (Stringaris, Zavos, Leibenluft, Maughan, & Eley, 2012). Our findings suggest that the same may apply to manic symptoms in childhood. Taking account of the heterogeneity reflected in the two dimensions could help refine the search for underlying mechanisms in BD. If further validated, such a dimensional approach to symptoms may be useful to clinicians. For example, treating clinicians may choose to target the symptoms of episodic undercontrol as opposed to the apparently more innocuous – or even beneficial – symptoms of episodic exuberance. As psychopathologists, we mainly study the negative outcomes of symptoms. Here, we show a striking example of symptoms that are associated with superior outcomes. Perhaps this is a more widespread phenomenon, with other apparently disadvantageous psychiatric symptoms persisting in the population because they are closely related to potentially beneficial traits (Williams and Taylor, 2006). Highlighting the positive correlates may make the adverse symptoms more tolerable to the individuals and their families. Having said that, we should stress again that the degree to which the mania symptoms reported in this study are part of a spectrum with manic episodes of people with bipolar disorder has yet to be established.

Our study has a number of strengths, such as the use of a large epidemiological study, the assessment of a broad range of manic symptoms by two reporting sources, and the use of standardized cognitive measures. Our study also has limitations. First, the design of our study does not allow us to determine direction of effect between manic symptoms and cognition. Intellectual endowment and response inhibition are commonly thought of as stable traits, in which case they would precede symptoms of mania. However, response inhibition may be impaired during a manic episode and positive affect has been reported to boost IQ (Donnelly et al., 1982). A second limitation is that we use the WISC to examine superior verbal performance in our study. This has the advantage of being a widely used, standardized and well-validated measure of verbal intelligence, which has previously been implicated in psychopathology (Hurlow & MacCabe, 2011). However, future studies in this area should ideally use a broad set of measures of verbal performance that also capture aspects of creativity, capacity for use of abstract or figurative language and generation of novel semantic constructs (Giora, 2003, Stringaris and Giora, 2011). A third limitation is that the design of our study does not allow us to prove that the symptoms reported here are of the same nature as manic symptoms reported by patients in clinical settings and we note that patients with BD who present to clinics may have impaired IQ and that it may further decrease with increasing number of episodes (Hellvin et al., 2012).

## Key points

1. Bipolar may be a heterogeneous construct, with some people showing superior premorbid adjustment and intelligence.
2. This heterogeneity may be due different symptom dimensions underlying mania.
3. In a community sample, we found support for the hypothesis that an exuberant dimension of symptoms characterized by cheerfulness and increased energy was positively and significantly associated with verbal IQ.
4. An undercontrol dimension, characterized by distractibility, irritability and risk taking was associated with poor response inhibition.
5. The two dimensions may have different aetiological underpinnings. Clinicians should be alert to the possibility that some manic symptoms may be related with higher than average abilities.
6. Standardized (beta) regression coefficients and their standard errors are presented from gender-adjusted models with either verbal IQ or performance IQ as outcomes, unadjusted or adjusted for sensation seeking with exuberance and undercontrol as predictors. ****p* < .001; ***p* <.01; **p* <.05; ns = non significant at *p* < .05.

## Supporting Information

Table S1. Description of the subsamples reporting manic symptoms by either parent- or self-report. [PDF file].
