# Supplementary material for: Dimensions of manic symptoms in youth: psychosocial impairment and cognitive performance in the IMAGEN sample
Source: J Child Psychol Psychiatry. 2014 May 28;55(12):1380–9. doi: 10.1111/jcpp.12255 (PMC4167034; doi:10.1111/jcpp.12255)
Supplement: Supplementary file 1 — Table S1. Description of the subsamples reporting manic symptoms by either parent- or self-report. [PDF file]. [file jcpp0055-1380-sd1.pdf]

Online supplementary information for: Dimensions of manic symptoms in youth: psychosocial impairment and cognitive performance in the IMAGEN sample by Stringaris, A., Ryan-Castellanos, N., Banaschewski, T. et al.

Table S1. Description of the subsamples reporting manic symptoms by either parent- or self-report.

	Age Mean in years (SD)	Gender (% male)	Ethnicity (% Caucasian)	Any disorder (%)	Emotional Disorder (%)	Conduct Disorder (%)	Oppositional defiant disorder (%)	Attention Deficit Hyperactivity Disorder (%)	Verbal IQ Mean (SD)	Performance IQ Mean (SD)
Self report (N=986)	14.4 (0.43)	47.6	90.2	11.9%	7.3%	0.9%	1.0%	3.0%	112.5 (14.8)	108.1 (14.5)
Parent report (N=699)	14.4 (0.42)	43.6	88.8%	15.7%	8.1%	2.3%	1.6%	2.0%	110.9 (15.5)	106.7 (14.6)
